# Neck circumference as a measure of neck fat and abdominal visceral fat in Chinese adults

**DOI:** 10.1186/1471-2458-14-311

**Published:** 2014-04-04

**Authors:** Hong-Xing Li, Fen Zhang, Dong Zhao, Zhong Xin, Shu-Qin Guo, Shu-Mei Wang, Jian-Jun Zhang, Jun Wang, Yan Li, Guang-Ran Yang, Jin-Kui Yang

**Affiliations:** 1 Department of Endocrinology, Beijing Tongren Hospital, Capital Medical University, Beijing 100730, China; 2 Department of Endocrinology, Baoding No.1 Central Hospital, Baoding, Hebei 07100, China; 3 Department of Endocrinology, Luhe Hospital, Capital Medical University, Beijing 101149, China; 4 Beijing Key Laboratory of Diabetes Research and Care, Beijing 100730, China

**Keywords:** Central obesity, Visceral adipose tissue, Neck circumference, Sagittal abdominal diameter, Computer tomography

## Abstract

**Background:**

Visceral adipose tissue (VAT) is a unique pathogenic fatty deposit, in that it is closely correlated with risk of cardiovascular diseases. The present study is to investigate the usefulness of neck circumference (NC) to indicate VAT.

**Methods:**

Participants aged 35 to 75 years who had taken abdomen and neck computer tomography (CT) examination were included in this study. Neck adipose tissue, abdominal VAT and subcutaneous adipose tissue (SAT) areas, as well as sagittal abdominal diameter (SAD) were measured by CT. Body anthropometrics and metabolic parameters including blood glucose, lipid profiles and blood pressure were also measured.

**Results:**

A lower abdomen CT examination was carried out on a total of 177 patients (87 male and 90 female) with a mean age of 59 years. Of the 177 participants, 15 men and 15 women also took a neck CT examination. With a comparable age and BMI, neck adipose area was correlated with abdominal VAT area significantly in men (r = 0.57, p = 0.028) and women (r = 0.53, p = 0.041). NC is positively correlated with VAT both in men (r = 0.49, p < 0.001) and women (r = 0.25, p = 0.012). Meanwhile, SAD is the best predictor for visceral fat both in men (r = 0.83, p < 0.001) and women (r = 0.73, p < 0.001). Body mass index (BMI), waist circumference (WC), and waist to height ratio (WHtR) correlated significantly with VAT both in men and women (r = 0.68, 0.42, 0.46 in men and 0.50, 0.23, 0.39 in women, p < 0.001), while waist hip ratio (WHR) displayed the weakest least correlation in men (r = 0.32, p = 0.001) and no correlation in women (r = 0.08, p = 0.442). Additionally, BMI was more strongly correlated with VAT than NC in both sexes (both p < 0.01).

**Conclusion:**

Significant correlation between NC and VAT was present in Chinese men and women, which may be accounted by the fact that neck fat area is significantly correlated with abdominal VAT. Meanwhile, SAD is the best predictor for visceral fat in the Chinese population.

## Background

An upper-body distribution of fat, especially with increased visceral fat, is more predictive of the metabolic complications of obesity than is body mass [1]. Results from the Framingham Heart Study indicated that upper-body subcutaneous fat, measured as neck circumference (NC) may be a unique, pathogenic fat deposit [2]. In a previous study of 3,182 diabetic patients, NC was positively related with body mass index (BMI), waist circumference (WC) and metabolic syndrome in Chinese individuals with type 2 diabetes [3]. However, the relationship between NC and visceral adipose tissue (VAT) calculated by images of computer tomography (CT) has not been extensively evaluated in diverse healthy and clinical populations.

Regional adipose tissue parameters, especially visceral tissue mass are more closely associated with physiological and pathological processes than total adipose tissue mass [4]. There is a close correlation between an increased quantity of VAT and cardiovascular diseases (CVD) [5]. However, application is limited by the radiation exposure associated with CT and by the relatively high cost of magnetic resonance imaging (MRI) analysis. Therefore, some substitute index of VAT, such as chubby cheeks and NC, will be more practical to predict the risk of CVD in primary care clinics. To date, there have been no published studies on the relationship between NC and VAT in a Chinese population. In this study we investigate whether NC is associated with VAT in patients who underwent CT scanning. To our knowledge, this is the first paper to compare neck fat with VAT by quantified radiographic measurements.

## Methods

### Subjects

Data were collected between September 2011 and May 2012 in 177 patients who took lower abdomen and neck CT examinations. All the patients were Chinese aged 35 to 75 years. Patients who met any of the following criteria were excluded from the study: abdominal disease that might affect the distribution of fat; thyroid disease; history of using cortical steroids; current treatment with statins or glucocorticoid; Cushing’s syndrome or other disorders of the pituitary or adrenal glands; and recent, substantial weight loss or weight gain. The study was approved by the Medical Ethics Committee of Beijing Tongren Hospital, Capital Medical University, and all participants provided a written informed consent.

### Anthropometric measurements

Anthropometric data including body weight, height, neck, hip and WC in centimeters of all participants in light clothing were evaluated by trained doctors using standard protocols. Body weight was measured using standardized equipment to the nearest 100 g, and height was measured by a stadiometer. WC was measured to the nearest centimeter between the iliac crest and the lower rib. Hip circumference was measured to the nearest centimeter, and BMI was calculated from the formula: weight (kg)/height^2^ (m^2^). Waist to hip ratio (WHR) and waist to height ratio (WHtR) were also calculated. NC was measured by placing a non-elastic flexible tape just below the laryngeal prominence and applied perpendicular to the long axis of the neck (to the nearest 0.5 cm) [3]. Sagittal abdominal diameter (SAD) was determined via an electronic measurement using the umbilical CT image with the cursor extending from skin to skin through the center of the abdomen in the anterior-posterior direction.

### Abdominal and neck fat determination

The technique used for adipose tissue area measurements on a single CT cross-sectional image (5 mm) has been previously described [6]. Participants were examined with a 64-detector row CT scanner (Philips Brilliance 64, Best, the Netherlands). Participants lay in a supine position with arms overhead to obtain a cross-sectional image examined at C4-5 and the L4-5 intervertebral space (120kVp, 338 Ma, gantry rotation time, 740 ms). Neck adipose tissue, abdominal VAT and subcutaneous adipose tissue (SAT) areas (cm^2^) were measured with commercially available CT software, ANALYZE Direct 10.0 (Mayo Clinic, Rochester, MN), and electronically determined by image display window width of -195 to -45 Housfield units. The muscular walls separating visceral fat from the subcutaneous compartment were traced manually.

### Statistical analysis

Statistical analyses were conducted separately for men and women because of known differences in adiposity. The data for the continuous variables with a normal distribution is expressed as means ± SDs, and the data for the continuous variables without a normal distribution is expressed as medians with interquartile ranges. The Student’s t-test or the Mann–Whitney test were used, as appropriate, to determine differences in continuous variables. Pearson’s correlation coefficients between the abdominal adipose tissue compartments (SAT and VAT) and the anthropometric measures (BMI, WC, WHR, WHtR, NC, SAD) were calculated. The independent relationship between anthropometric measures and abdominal adipose tissue compartment was examined by multiple regression analysis adjusted by age and determination of the standardized correlation coefficients. p value of less than 0.05 were considered statistically significant. All statistical analysis was performed using SPSS 18.0 software (Statistic Package for the Social Sciences). Graphs described by Origin software 7.5 (OriginLab Corp., Northampton, MA. USA).

## Results

### Characteristics of the participants

The study contained 177 subjects (87 male and 90 female) with a mean age of 59. Age, blood glucose, blood pressure, lipid profile, BMI were not significantly different between the male and female groups. As expected, women had a significantly greater percentage of SAT but a lower WC, WHR, NC, SAD and VAT than did the men. VAT area and NC were significantly different between men and women (123.7 ± 55.1 cm^2^ vs 104.5 ± 45.4 cm^2^ for VAT area, p < 0.01 and 39 (31.5-47) cm vs 35 (30–45) cm for NC, p < 0.001, respectively) (Table 1).

**Table 1:** Clinical and biochemical characteristics of the study subjects

	**Abdomen CT participants**			**Neck CT participants**		
	**Male**	**Female**	**P -value**	**Male**	**Female**	**P -value**
n	87	90		15	15	1
Age (years)	58.7 ± 9.3	58.8 ± 10.5	0.449	50	50	1
Metabolic parameters						
SBP (mmHg)	138.7(100,200)	132.7(90,190)	0.159	133(122,180)	125(100,155)	0.166
DBP (mmHg)	84.5(60,120)	80.9(60,110)	0.102	85(70,110)	79(65,95)	0.112
BG (mmol/l)	5.4(4.8,7.1)	5.2(4.5,6.9)	0.426	---	---	---
TG (mmol/l)	1.65(0.38,8.1)	1.34(0.53,4.75)	0.359	---	---	---
CHO (mmol/l)	4.86(1.32,8.01)	5.38(0.8,9.35)	0.033	---	---	---
VLDL (mmol/l)	0.79 ± 0.62	0.65(0.12,2.16)	0.217	---	---	---
LDL (mmol/l)	2.92 (0.32,6.17)	3.24(0.49,6.65)	0.083	---	---	---
HDL (mmol/l)	1.23(0.51,5.17)	1.30(0.12,4.71)	0.495	---	---	---
Body composition parameters						
BMI (kg/m^2^)	25.7 ± 3.8	25.0 ± 3.7	0.265	26.2 ± 0.5	26.2 ± 0.6	0.971
WC (cm)	95.3(71,120)	91.3(64,112)	0.003	95.1 ± 4.3	90.2 ± 3.9	0.003
WHR	0.96 ± 0.06	0.92 ± 0.06	0.000	0.93 ± 0.05	0.89 ± 0.04	0.612
WHtR	0.56 ± 0.06	0.57 ± 0.06	0.164	0.54 ± 0.08	0.57 ± 0.05	0.386
NC (cm)	39.0 (31.5,47)	35.0(30,45)	0.006	40(38,45)	35(33,39)	0.001*
SAD (cm)	22.4(15.7,32.2)	20.8(14.7,26.3)	0.000	21.1(18,26.9)	19.0(16.5,21.5)	0.002*
CT image						
SAT area (cm^2^)	156.6 ± 62.0	196.7 ± 73.6	0.000	170.5 ± 26.7	225.3 ± 41.2	0.000
VAT area (cm^2^)	123.7 ± 55.1	104.5 ± 45.4	0.012	173.7 ± 47.9	119.6 ± 27.0	0.001
TAT area (cm^2^)	280.3 ± 107.1	301.1 ± 104.1	0.191	344.2 ± 60.7	344.9 ± 43.0	0.969
VS ratio	1.1(0.32,2.61)	1.2(0.19,4.69)	0.268	1.0 ± 0.3	0.6 ± 0.2	0.000
Neck adipose area (cm^2^)	---	---	---	26.4 ± 7.8	34.1 ± 5.5	0.004

Values are expressed as means (medians) ± SDs (range), *P value refers difference between male and female.

SBP: systolic blood pressure; DBP, diastolic blood; BG: blood glucose; TG: triglycerides; CHO: Total cholesterol; VLDL: very low density lipoprotein; LDL: low density lipoprotein; HDL: High density lipoprotein; BMI: body mass index; WC: waist circumference; WHR: Waist to hip ratio; WHtR: Waist to height ratio; NC: neck circumference; SAD: sagittal abdominal diameter; SAT: subcutaneous adipose tissue; VAT: visceral adipose tissue; TAT: total adipose tissue; VS ratio: visceral to subcutaneous adipose ratio.

### Neck and abdominal adiposity measurements

**Figure 1:**
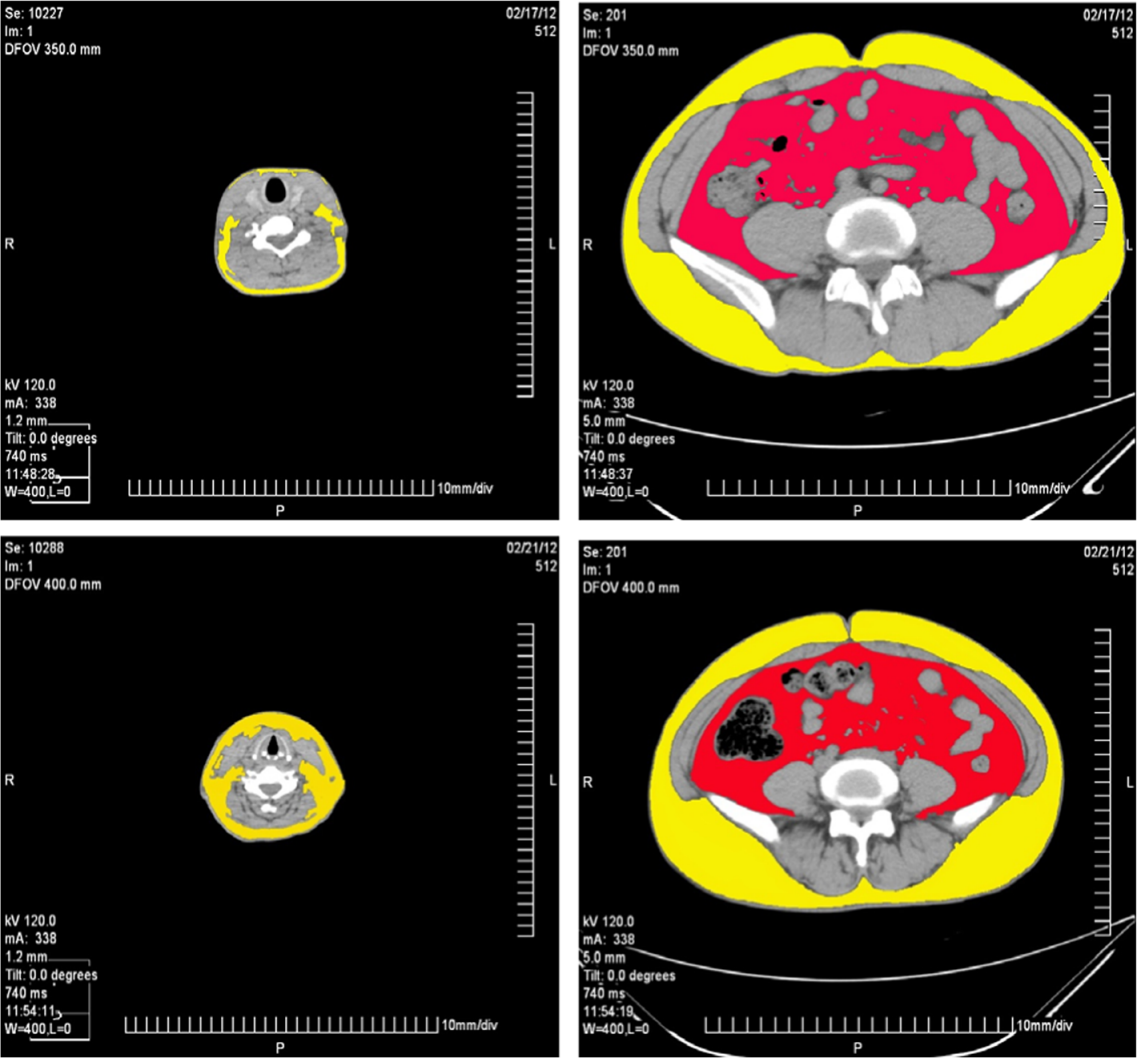
**Comparison of the cross-sectional images of C4-5 and L4-5 adipose area in a male and a female participants.** Yellow area refers to subcutaneous fat and green area refers to neck fat or visceral fat. With a comparable abdominal subcutaneous and visceral adipose tissue, the male participant has a less neck adipose than the female.

**Figure 2:**
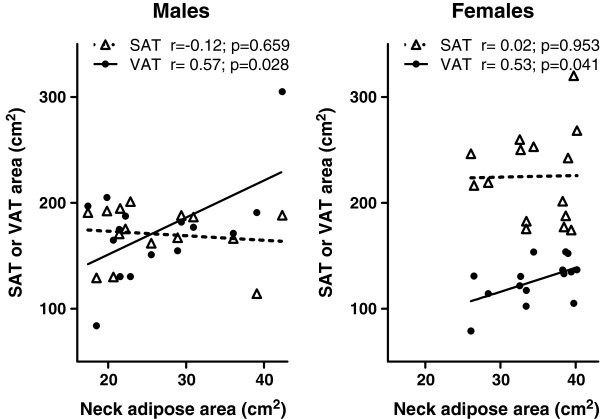
**The relationship between neck adipose tissue and SAT and VAT area in males and females.** Neck adipose area is correlated with abdominal VAT but not SAT significantly both in the male and female group.

Among the 177 subjects, 15 men and 15 women matched for age and BMI took both neck and abdominal adiposity measurement. Age, blood pressure, lipid profile, WHR, WHtR and BMI are insignificantly different between male and female groups (Table 1). Different fat distributions are shown in Figure 1. Compared with women, men tended to have a larger neck circumference, less neck adipose tissue and more VAT deposit. With a comparable total abdominal adipose tissue, the male participants had less neck adipose than did the female (Table 1). Neck adipose area was correlated with abdominal VAT area significantly in the male group (r = 0.57, p = 0.028) and the female group (r = 0.53, p = 0.041). However, no correlation was found between neck adipose area and abdominal SAT area (Figure 2).

### Meanings of neck circumference

As showed in Table 2, Partial correlation coefficient was used to examine the independent correlation of NC with VAT after controlling for the effects of age. NC is more closely associated with abdominal SAT than VAT both in men (r = 0.59, p < 0.001 vs. r = 0.49, p < 0.001) and women (r = 0.41, p < 0.001 vs. r = 0.25, p = 0.012). These data also indicate a significant correlation between NC and VAT both in men and women.

**Table 2:** Univariate associations between body compositions and SAT/VAT in males and females

	**Male (n = 87)**		**Female (n = 90)**	
	**SAT**	**VAT**	**SAT**	**VAT**
NC	0.59***	0.49***‡	0.41***	0.25*†
BMI	0.79***	0.68***	0.64***	0.50***
WC	0.40**	0.42***	0.34***	0.23*
WHtR	0.49***	0.46***	0.54***	0.39***
WHR	0.36**	0.32**	0.08	0.09
SAD	0.74***	0.83***	0.72***	0.73***

R is Pearson correlation coefficient. ***P < 0.001; **P < 0.01; *P < 0.05.

SAT: subcutaneous adipose tissue; VAT: visceral adipose tissue; BMI: body mass index; WC: waist circumference; WHtR: Waist to height ratio; WHR: Waist to hip ratio; NC: neck circumference; SAD: sagittal abdominal diameter.

‡: in Male, Pearson correlation coefficient of NC and VAT vs. BMI and VAT, t = 3.12, p < 0.01.

†: in Female, Pearson correlation coefficient of NC and VAT vs. BMI and VAT, t = 2.18, p < 0.01.

### Correlations between body fat and other anthropometric measurements

Table 2 also shows the partial correlation coefficient analysis for the association of the regional abdominal fat distribution, with other body anthropometric measures except NC, after adjustment for age. There is a strong relationship between anthropometric measures of SAD and BMI with VAT (r = 0.83 and 0.68 in male, 0.73 and 0.50 in female group separately, p < 0.001), moderate relationship between WC, WHtR with VAT(r = 0.42,0.46 in male and 0.23,0.39 in female, p < 0.001). Further compare the correlation coefficient on BMI and NC with VAT in both gender group shows the difference is significance (p < 0.01). Among the four measures, SAD is the best predictor for VAT (r = 0.83 and r = 0.73 for men and women, respectively) and SAT (r = 0.74 and r = 0.72 for men and women, respectively). BMI, WC, WHR and WHtR are more correlated with SAT than VAT significantly. In female group, WHR have significant correlation neither with VAT nor with SAT.

### Multiple linear regression for body compositions with VAT

The SAD was a significant predictor for visceral fat both in men and women group (Table 3), and BMI is superior to other indices in male group. Other anthropometrics such as WHtR, WC, NC and WHR are fail to entry the equation.

**Table 3:** Multiple linear regression analysis between body compositions and VAT adjusted by age

**Gender**	**Explanatory variables**	**Independent variable coefficients (β)**	**p-value**	**R-square**
Male (n = 87)	BMI	-2.003	0.048	0.674
	WC	-1.483	0.142	
	WHtR	1.277	0.085	
	WHR	0.333	0.740	
	NC	0.404	0.688	
	SAD	7.696	0.000	
Female (n = 90)	BMI	-1.254	0.214	0.528
	WC	-.742	0.460	
	WHtR	1.138	0.259	
	WHR	-1.189	0.238	
	NC	0.751	0.455	
	SAD	6.786	0.000	

Adjusted by age. BMI: body mass index; WC: waist circumference; WHtR: Waist to height ratio; WHR: Waist to hip ratio; NC: neck circumference; SAD: sagittal abdominal diameter.

## Discussion

Imaging methods such as CT and MRI are considered to be among the most accurate approaches for the in-vivo quantification of body composition. VAT assessed by single slice CT image at the umbilicus level or the lumbar vertebral 4th to 5th level were widely used in previous studies [7,8]. However, it is necessary to substitute VAT by a simple anthropometric measure to discern the high risk individuals in physical examination.

In the present study, we demonstrated that NC, an upper body fat depot, is positively associated with abdominal visceral fat in Chinese adults, especially in male individuals. Early in 2001, NC screening was performed in 979 overweight and obese patients [9]. A population-based epidemiological follow-up study showed that the measurement of NC could be useful for predicting insulin resistance [10]. The Framingham Heart Study of 3307 patients indicated that NC is associated with CVD risk factors even after adjustment for VAT and BMI [2]. But few studies on NC and visceral fat in Asian populations have been published. Our previous study showed that NC is a good predictor of metabolic syndrome in patients with type 2 diabetes [3]. The present study was to evaluate the usefulness of NC and SAD in predicting visceral obesity in Chinese by comparing NC and SAD with WC, BMI, WHR and WHtR.

The interesting finding from the present study is that NC, though difference in gender for body anatomic structure, correlates significantly with abdominal VAT, especially in males. It may account for the fact that neck fat area is associated with abdominal visceral fat in men and women. Our result is in consistence with previous study, which showed NC is strongly associated with VAT both in man and woman [3]. Whittle AT et al. previously measured the fat and tissue volumes in the necks of 10 non-obese men and 10 women at the age 37 and BMI 25 kg/m2 by MRI [11]. They demonstrated that men tended to have larger soft tissue and great proportion of neck fat inside the mandible anterior segment at the palatal level around airway. This paradoxical aspect may because the different protocol designs on image scanning location, age and BMI. Especially, adipose distribution varied from sex, age, ethnic [12], and sex effects of VAT diminish with aging [13].

In addition to NC, this study also showed correlation between other anthropometric measures and VAT. SAD correlated more strongly with VAT than SAT irrespective of age, sex and the degree of obesity versus other anthropometric measures. Just as anticipated, in Chinese adults, SAD had a strongest correlation with VAT than SAT, which coincides with the findings of the study in Korea [14]. In our study, we obtained the data by calculating the SAD on CT slides. This new anthropometric measure is worthy of much attention for its potential to be implemented as a large scale epidemiological screening of central obesity in China. As referred by other authors [15], SAD is the best predictor for VAT in Chinese populations. BMI and WC are widely used indices for obesity in clinical work as recommended by the WHO. Moreover, WC is a better predictor for VAT than BMI [16]. The slope of the linear relationship of WC and VAT varied from 0.49 to 0.77 for the impact of racial/ethnic, age and sex –differences in previous papers [12,17,18]. Above all, measurement of WC in the obese and eldly persons at the umbilicus may be directed downward because of the excessive curvatures of the abdominal wall. It is necessary to apply a suitable ruler or apparatus to measure SAD supplely and accurately enough for community screening projects, just as described by Risérus U [19].

WHtR and WHR have been reported by authors to closely associated with metabolic risk factors and coronary heart disease (CHD) in Chinese populations [20-22]. Our present study shows that WHR and WHtR both correlate with VAT. This reminds us that WC may be less stable and may fluctuate under different circumstances; it may be influenced by expiration, position and abdominal shape. Though WHR is the weakest predictor for VAT, it may not reflect its validity on predicting diabetes and cardiovascular disease [15,23]. Visceral obesity is inversely related with cardiovascular outcomes. The clinical performance in different genders and ethnicities may vary. A recent paper on 2477 men and 3107 women in Shanghai suggests that the independent use of BMI, WC, WHR, or WHtR may not be an effective tool to predict metabolic factors and related chronic diseases in Chinese adults [24]. Here we propose that NC and SAD should be considered new measures and carried out in prospective studies to verify their validity as screening methods for cardiovascular risks.

Our study has some limitations, such as the small sample size and the fact that our subjects were patients in a clinical study. Our findings are yet to be verified in a community setting. This is merely a preliminary study that warrants further research. The conclusion that NC is a better clinical predictor of VAT than is general body obesity may be extended to wider populations with some confidence.

## Conclusion

Our data indicates that there is an association between neck circumference and neck fat with visceral fat in Chinese adults. Among anthropometric measures, SAD is the best surrogate index to predict visceral fat. BMI, WC and WHtR are more strongly associated with SAT than with VAT. WHR is the weakest predictor for VAT both in males and females. Such analyses are helpful for physicians who even without immediate access to facilities for visceral fat detection can depend on simpler clinical features to raise their suspicion of the disease.

## Competing interest

The authors declare that they have no competing interests.

## Authors’ contributions

Researched data, wrote manuscript, and contributed to the discussion: LHX, ZF, XZ. Researched data: GSQ, WSM, ZJJ, WJ, LY. Contributed to the discussion: ZD, YGR. Designed, Researched data, wrote manuscript, and contributed to the discussion: YJK. All authors read and approved the final manuscript.

## Pre-publication history

The pre-publication history for this paper can be accessed here:

http://www.biomedcentral.com/1471-2458/14/311/prepub
